# Early overnutrition sensitizes the growth hormone axis to the impact of diet-induced obesity via sex-divergent mechanisms

**DOI:** 10.1038/s41598-020-70898-y

**Published:** 2020-08-17

**Authors:** M. A. Sanchez-Garrido, F. Ruiz-Pino, A. I. Pozo-Salas, J. M. Castellano, M. J. Vazquez, R. M. Luque, M. Tena-Sempere

**Affiliations:** 1 Maimónides Institute of Biomedical Research of Córdoba (IMIBIC), Córdoba, Spain; 2 Department of Cell Biology, Physiology and Immunology, Faculty of Medicine, University of Córdoba, Avda. Menéndez Pidal s/n., 14004 Córdoba, Spain; 3 Reina Sofia University Hospital, Córdoba, Spain; 4 CIBER Fisiopatología de La Obesidad y Nutrición (CIBEROBN), Instituto de Salud Carlos III, 14004 Córdoba, Spain; 5 Institute of Biomedicine, University of Turku, 20520 Turku, Finland

**Keywords:** Physiology, Endocrinology

## Abstract

In addition to its essential role in the physiological control of longitudinal growth, growth-hormone (GH) is endowed with relevant metabolic functions, including anabolic actions in muscle, lipolysis in adipose-tissue and glycemic modulation. Adult obesity is known to negatively impact GH-axis, thereby promoting a vicious circle that may contribute to the exacerbation of the metabolic complications of overweight. Yet, to what extent early-overnutrition sensitizes the somatotropic-axis to the deleterious effects of obesity remains largely unexplored. Using a rat-model of sequential exposure to obesogenic insults, namely postnatal-overfeeding during lactation and high-fat diet (HFD) after weaning, we evaluated in both sexes the individual and combined impact of these nutritional challenges upon key elements of the somatotropic-axis. While feeding HFD per se had a modest impact on the adult GH-axis, early overnutrition had durable effects on key elements of the somatotropic-system, which were sexually different, with a significant inhibition of pituitary gene expression of GH-releasing hormone-receptor (GHRH-R) and somatostatin receptor-5 (SST5) in males, but an increase in pituitary GHRH-R, SST2, SST5, GH secretagogue-receptor (GHS-R) and ghrelin expression in females. Notably, early-overnutrition sensitized the GH-axis to the deleterious impact of HFD, with a significant suppression of pituitary GH expression in both sexes and lowering of circulating GH levels in females. Yet, despite their similar metabolic perturbations, males and females displayed rather distinct alterations of key somatotropic-regulators/ mediators. Our data document a synergistic effect of postnatal-overnutrition on the detrimental impact of HFD-induced obesity on key elements of the adult GH-axis, which is conducted via mechanisms that are sexually-divergent.

## Introduction

Growth hormone (GH) plays a pivotal role in longitudinal growth during development^1^. In addition, GH also exerts important actions on metabolic processes by promoting anabolic effects in the muscle and catabolic actions in the adipose tissue^2,3^. It is well established that adult GH deficiency causes alterations in body composition and lipid metabolism^4–7^. Adult GH deficiency is usually accompanied by increased abdominal fat accumulation, which is attenuated after GH replacement^8–11^. It is also known that abdominal obesity negatively affects GH secretion. This effect has been documented in obese humans and rodents, where spontaneous, as well as stimulated, GH secretion is markedly reduced^12–17^. Due to the lipolytic effect of GH on the adipose tissue, it has been suggested that such obesity-related disruption of GH secretion may promote a vicious cycle that might enhance adiposity and exacerbate the metabolic comorbidities linked to obesity^18^. Interestingly, weight loss in obese subjects reverses the decline in GH levels and improves stimulated GH output^19^, suggesting that the metabolic disturbances associated to weight gain may be responsible for the blunted basal and stimulated GH secretion in obesity.

The underlying mechanisms for the reduction in circulating GH levels in obesity are complex and not fully understood. An array of metabolic hormones and signals has been shown to alter GH output by acting at hypothalamic and/or pituitary levels. Several studies have provided evidence of the detrimental impact of increased circulating levels of insulin-like growth factor-1 (IGF-1), glucose, insulin, free fatty acids (FFA) and other metabolic factors on the GH axis^12,20,21^. Of note, plasma levels of all these metabolic factors are commonly elevated in obesity and, therefore, may ultimately contribute to obesity-associated GH deficiency.

Over the last decades, compelling evidence has suggested that early perturbations of the nutritional environment (e.g., during the perinatal period) might permanently influence metabolic health and other physiological parameters in adulthood^22^. Epidemiological and experimental studies have revealed a strong association between early nutritional alterations and the incidence of obesity and its related comorbidities later in life^23–26^. These findings suggest that the adaptive changes that occur during early stages of development may have a durable impact on key metabolic pathways that may predispose to metabolic abnormalities in subsequent stages of development. In addition, it has been suggested that early overfeeding may sensitize for the deleterious effects of a high-fat diet (HFD) in adulthood. In line with this hypothesis, our group and others have recently reported that early postnatal overnutrition exacerbates HFD-induced metabolic disturbances in adult life^27–31^. Interestingly, recent studies have also documented that early overnutrition may compromise the correct functioning of various neurohormonal axes in adulthood^27,32,33^, including the GH axis^34^. In this regard, it has been shown that early overnutrition increases somatic growth during development by altering hypothalamic GH-releasing hormone (GHRH) and pituitary GH expression in mice, changes that persisted in adult life^34^. However, whether early overfeeding may sensitize the GH axis for the deleterious effects of HFD in adulthood remains to date unexplored. Yet, such possibility is of considerable translational interest, given the escalating trends of child obesity^35^.

In the above context, the present study aimed at characterizing the potential impact of early postnatal overnutrition on the susceptibility of the GH axis to the impact of HFD-induced obesity in adult life, by means of the analysis of key hypothalamic, pituitary and liver components of the somatotropic axis, as well as relevant circulating hormones, in male and female rats subjected to these two obesogenic insults, with special attention being paid to the identification of potential sex-dependent differences in the pathophysiological mechanisms underlying the eventual alterations of the GH axis in such conditions.

## Results

Analyses of the elements of the somatotropic axis, including circulating levels of GH and IGF-1, as well as the gene expression levels of key factors at the hypothalamus [GHRH, somatostatin (SRIF), Neuropeptide-Y (NPY) and ghrelin], pituitary [GH, somatostatin receptors (SSTs), Leptin-receptor (Lep-R), Insulin-receptor (Ins-R), ghrelin, ghrelin-receptor (GHS-R), and Ghrelin-O-Acyl Transferase (GOAT)] and liver [GH-receptor (GH-R) and IGF-1], were implemented in serum and tissue samples from cohorts of male and female rats, generated in the context of large longitudinal studies addressing the impact of sequential obesogenic insults, namely postnatal overfeeding (SL) and exposure to HFD after weaning, on various reproductive and metabolic parameters. While metabolic characterization of these models was partially reported in previous publications of our group^27,28^, key metabolic parameters are summarized also here and presented in Supplemental Figure S3, for reference purposes.

### Metabolic profiles of adult male and female rats after early overnutrition or/and HFD

**Figure 1:**
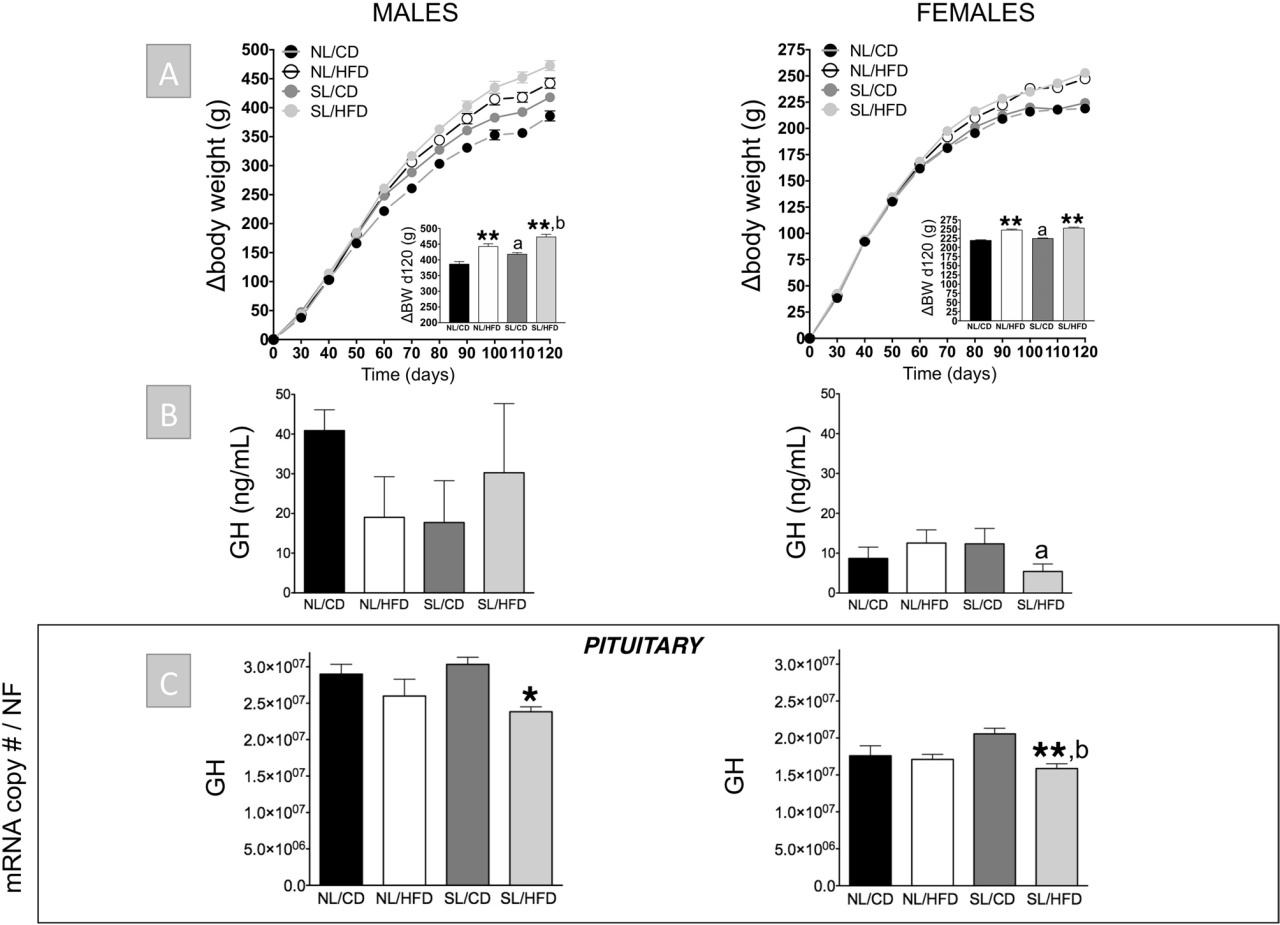
Cumulative delta body weight (BW) curves between PND 23 and 120 (**A**; the insets represent the absolute BW gain between PND 23 and 120), circulating GH levels (**B**) and pituitary GH mRNA levels (**C**) from male (*left panels*) and female rats (*right panels*) subjected or not to overfeeding during lactation (SL vs. NL) and/or HFD after weaning (HFD vs. CD). mRNA copy numbers were determined by qPCR and adjusted by a Normalization Factor (NF) in each sample obtained from the expression levels of three housekeeping genes (*β-actin*, *Hprt* and C*yclophilin A*). Data are presented as mean ± standard error of the mean (SEM), n = 8 per experimental group. Statistically significant differences were assessed by two-way ANOVA to analyze the effects of litter size and diet and their interactions. When significant differences were found, the data were further analyzed using Newman-Keuls tests to identify simple effects. *P < .05; **P < .01 effect of HFD; a, *P* < .05, effect of litter size; b, *P* < .05 interaction of litter size/HFD.

Postnatal overfeeding (SL) resulted in an increase in delta body weight (i.e., absolute BW gain) between PND 23 and 120 in both males and females (Fig. 1). However, representation of delta body weight curves during this period revealed that males were more sensitive to the body weight gain effect of SL than females, with an increase being detectable in SL/CD males versus NL/CD from PND 50 onwards (Fig. 1). Despite this obesogenic effect, SL per se did not significantly affect circulating leptin or insulin levels in either sex (NL/CD vs. SL/CD; Suppl. Fig. S3). However, SL males, but not females, exhibited elevated basal glucose levels as well as increased body length (NL/CD vs. SL/CD; Suppl. Fig. S3 and S4). After 12 weeks of exposure to HFD, NL males and females (NL/HFD) were heavier than their respective NL/CD controls, with enhanced delta body weight and increased daily energy intake (Fig. 1 and Suppl. Fig. S3). In addition, NL/HFD rats displayed elevated serum leptin levels, although such an increase reached statistical significance only in males (Suppl. Fig. S3). In contrast, basal insulin levels were oppositely affected in NL/HFD males (significant decrease) versus females (moderate, but not statistically significant increase; Suppl. Fig. S3). NL/HFD males also showed increased circulating glucose levels and body size as compared to control (NL/CD) males, while no significant differences in basal glucose levels or body length were detected between NL/CD and NL/HFD females (Suppl. Fig. S3 and S4). Finally, the combination of both obesogenic factors (SL/HFD) caused the highest increase in body weight as well as basal leptin and glucose levels in both sexes (Fig. 1 and Suppl. Fig. S3), while further enhancement of body length by SL/HFD was only observed in males (Suppl. Fig. S4). Moreover, no significant differences were detected in circulating insulin levels in SL/HFD male and female rats versus their corresponding controls (Suppl. Fig. S3).

### Impact of early overnutrition on the GH axis in adult male and female rats

**Figure 2:**
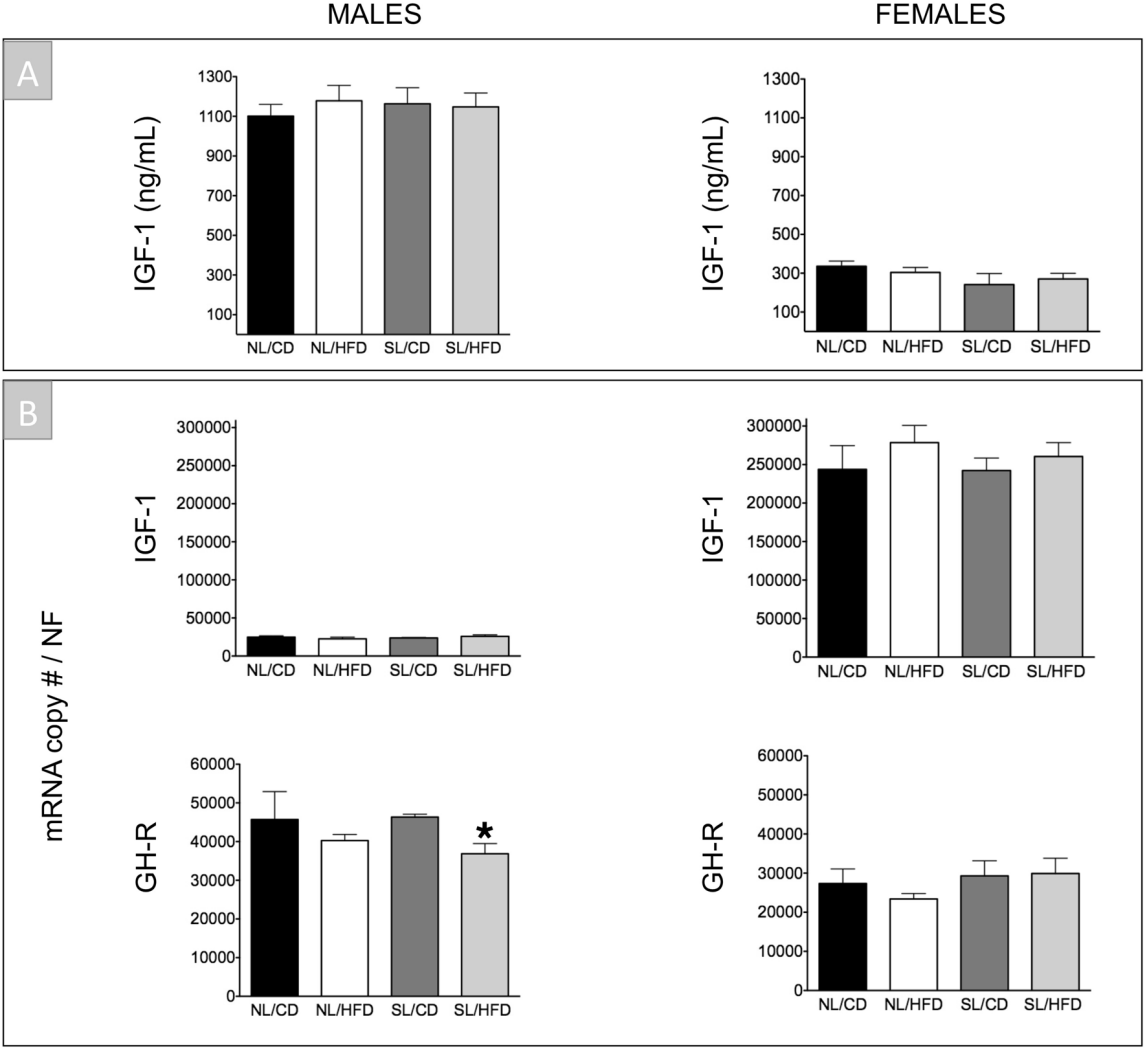
Circulating IGF-1 levels (**A**) and liver IGF-1 and GH-R mRNA levels (**B**) from male (*left panels*) and female rats (*right panels*) subjected or not to overfeeding during lactation (SL vs. NL) and/or HFD after weaning (HFD vs. CD). mRNA copy numbers were determined by qPCR and adjusted by a Normalization Factor (NF) in each sample obtained from the expression levels of three housekeeping genes (*β-actin*, *Hprt* and C*yclophilin A*). Data are presented as mean ± standard error of the mean (SEM), n = 8 per experimental group. Statistically significant differences were assessed by two-way ANOVA to analyze the effects of litter size and diet and their interactions. When significant differences were found, data were further analyzed using Newman-Keuls tests to identify simple effects. *P < .05 effect of HFD.

**Figure 3:**
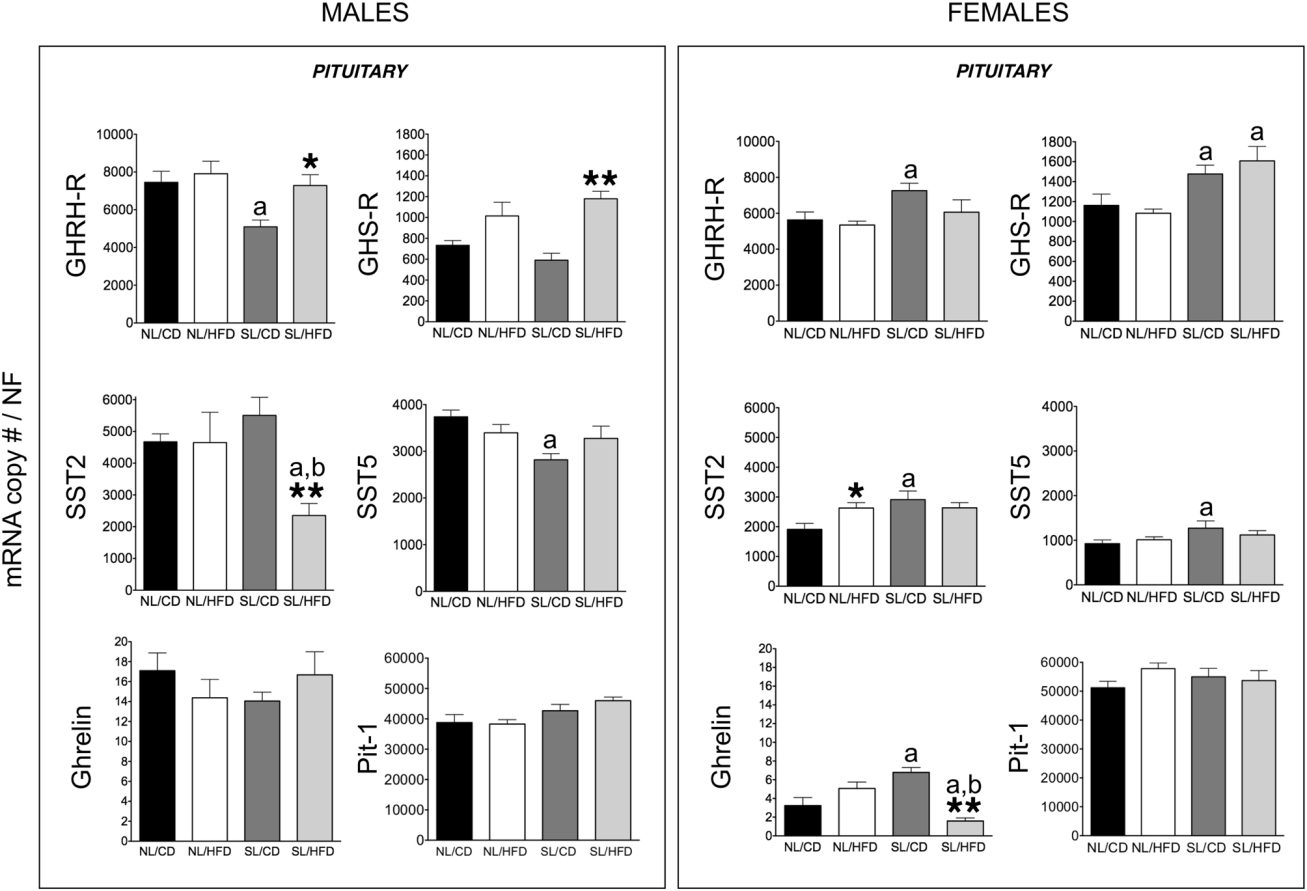
Pituitary GHRH-R, GHS-R, SST2, SST5, ghrelin and Pit-1 mRNA levels in male (*left panels*) and female rats (*right panels*) subjected or not to overfeeding during lactation (SL vs. NL) and/or HFD after weaning (HFD vs. CD). mRNA copy numbers were determined by qPCR and adjusted by a Normalization Factor (NF) in each sample obtained from the expression levels of three housekeeping genes (*β-actin*, *Hprt* and C*yclophilin A*). Data are presented as mean ± standard error of the mean (SEM), n = 8 per experimental group. Statistically significant differences were assessed by two-way ANOVA to analyze the effects of litter size and diet and their interactions. When significant differences were found, the data were further analyzed using Newman-Keuls tests to identify simple effects. *P < .05; **P < .01 effect of HFD; a, *P* < .05, effect of litter size; b, *P* < .05 interaction of litter size/HFD.

**Figure 4:**
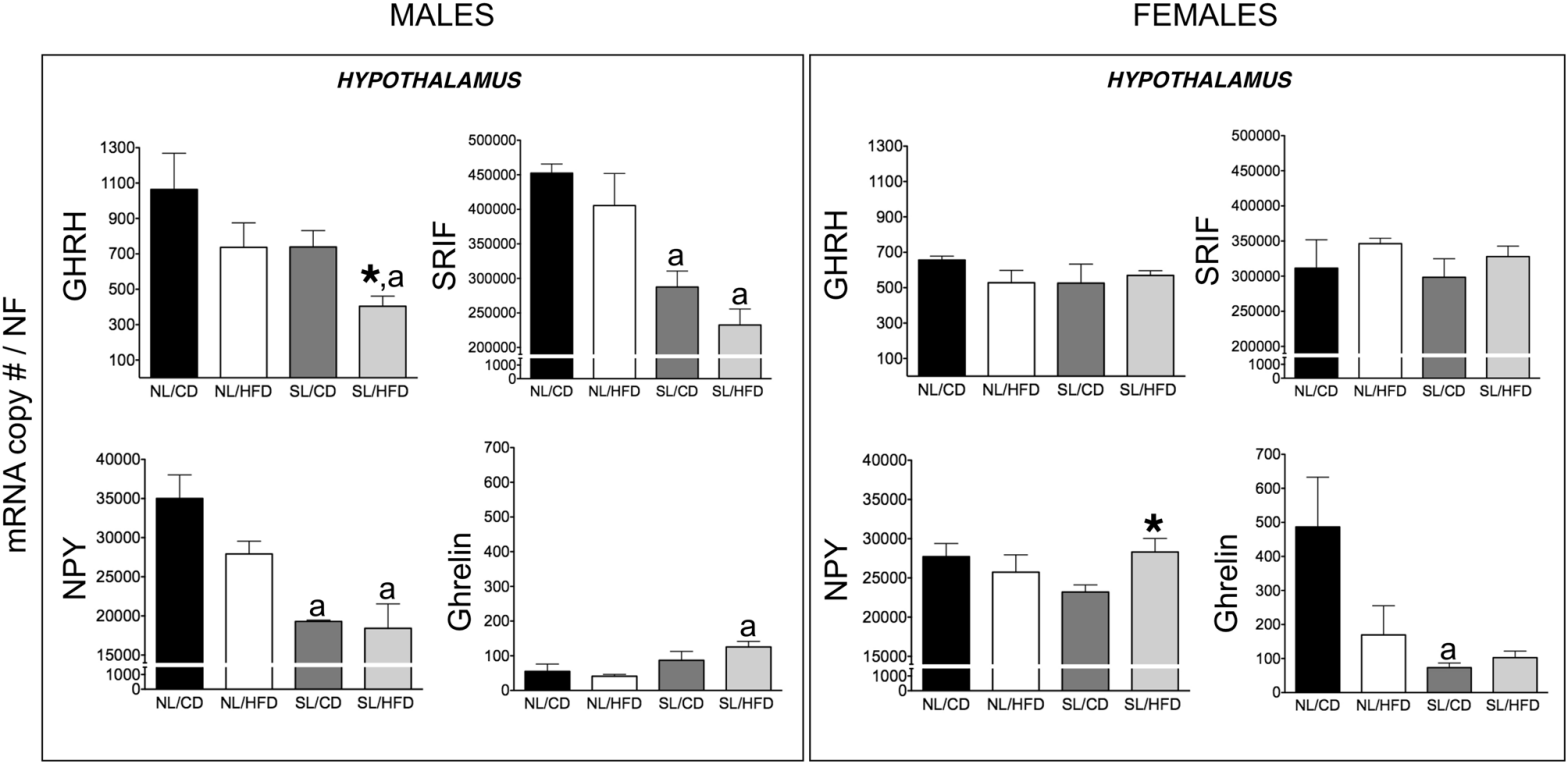
Hypothalamic GHRH, SRIF, NPY and ghrelin mRNA levels in male (*left panels*) and female rats (*right panels*) subjected or not to overfeeding during lactation (SL vs. NL) and/or HFD after weaning (HFD vs. CD). mRNA copy numbers were determined by qPCR and adjusted by a Normalization Factor (NF) in each sample obtained from the expression levels of three housekeeping genes (*β-actin*, *Hprt* and C*yclophilin A*). Data are presented as mean ± standard error of the mean, n = 5–6 per experimental group. Statistically significant differences were assessed by two-way ANOVA to analyze the effects of litter size and diet and their interactions. When significant differences were found, the data were further analyzed using Newman–Keuls tests to identify simple effects. *P < .05 effect of HFD; a, *P* < .05 effect of litter size.

Early overnutrition (SL) per se did not cause significant alterations in circulating GH levels compared with NL/CD controls in both sexes, with GH levels being consistently higher in male than in female groups, in line with previous references^36,37^. Accordingly, no changes were detected in pituitary GH mRNA levels between SL/CD and NL/CD male and female rats (Fig. 1). In good agreement, circulating IGF-1 levels (also higher in males than in females), as well as hepatic expression of IGF-1 and GH-R levels in SL/CD rats did not differ from those of the control NL/CD group in both sexes (Fig. 2). Yet, SL/CD males displayed a significant reduction in pituitary GHRH-R and SST5 mRNA levels compared to NL/CD controls (Fig. 3). In contrast, pituitary ghrelin, GHS-R and SST2 expression in SL/CD males were not significantly altered (Fig. 3). In contrast, SL/CD females displayed a significant increase in pituitary GHRH-R, GHS-R, SST2, SST5 and ghrelin mRNA levels versus control NL/CD rats (Fig. 3). In addition, early overfeeding did not alter pituitary leptin and insulin receptor expression in either sex, neither it affected the expression levels of Pit-1, SST1, SST3 or GOAT mRNAs (Fig. 3 & Suppl. Fig. S2 and S4), while SST4 expression was undetectable at the pituitary (*data not shown*). Interestingly, at the hypothalamic level, SL/CD males showed a significant reduction in SRIF and NPY expression versus NL/CD controls, which was not detected in females (Fig. 4). Hypothalamic GHRH mRNA levels were not altered by SL in both sexes, whereas hypothalamic ghrelin expression was significantly reduced by SL only in females (Fig. 4).

### Effect of HFD on the GH axis in adult male and female rats

At PND-120, male rats fed on HFD showed a trend for decreased circulating GH levels (*P* = 0.09; NL/CD controls vs. NL/HFD), without significant changes being detected on pituitary expression of GH (Fig. 1). In females, HFD alone did not alter serum GH levels or pituitary GH expression (Fig. 1). Similarly, no significant changes were detected in serum IGF-1 levels and hepatic IGF-1 and GH-R expression in NL/HFD males and females vs NL/CD controls (Fig. 2). In males, HFD did not significantly affect pituitary expression of GHRH-R, SST2, SST5 and ghrelin, but caused a non-significant increase in GHS-R mRNA levels (*P* = 0.07, Fig. 3). Likewise, NL/HFD females did not display significant alterations in pituitary GHRH-R, GHS-R, SST5 and ghrelin mRNA levels, but HFD caused a significant increase in pituitary SST2 expression (Fig. 3). Similar to SL, HFD did not alter the pituitary expression of the genes encoding leptin or insulin receptors in males or females, nor did it modify pituitary Pit-1, SST1, SST3 or GOAT mRNA levels (Fig. 3 & Suppl. Fig. S2 and S4), whereas SST4 mRNA expression was below the limit of detection at the pituitary in any of the experimental groups (*data not shown*). At the hypothalamic level, HFD failed to alter GHRH expression in either males or females (Fig. 4). In the same line, hypothalamic SRIF, NPY and ghrelin mRNA levels did not significantly differ between NL/CD and NL/HFD groups of both sexes (Fig. 4).

### Combined effect of early overnutrition and HFD on the GH axis in adult rats

The combination of both obesogenic factors, SL and HFD, caused a significant reduction in pituitary GH mRNA levels compared to their respective SL/CD controls in both males and females (Fig. 1). Such a reduction was accompanied by a non-significant decrease (> 50%) in circulating GH levels in females, whereas basal GH levels were not overtly affected by the combination of both factors in males (Fig. 1). Despite these alterations in pituitary GH expression and serum GH levels, no changes in liver IGF-1 expression or circulating IGF-1 levels were detected between SL/CD and SL/HFD rats in both sexes (Fig. 2). Yet, SL/HFD males showed a significant suppression of liver GH-R mRNA levels versus the control (SL/CD) group; an effect that was not detected in females (Fig. 2). Analyses of pituitary components of the GH axis in males revealed that the combination of both obesogenic insults significantly increased pituitary mRNA levels of GHRH-R and GHS-R as compared to SL/CD controls (Fig. 3). In addition, SL/HFD males showed a pronounced and significant reduction in pituitary SST2 mRNA levels, the major receptor that drives the inhibitory effects of SRIF on pituitary GH expression and release (Fig. 3). SL/HFD males also displayed increased pituitary expression of leptin and insulin receptors (Suppl. Fig. S2). In contrast, SL/HFD females did not display alterations in pituitary GHRH-R, GHS-R, SST2, leptin or insulin receptor mRNA levels versus the corresponding SL controls (Fig. 3 & Suppl. Figure 2). However, the combination of postnatal overnutrition and HFD in females caused a significant decrease in pituitary ghrelin expression, which was not detected in males (Fig. 3). In contrast, in SL/HFD male and female rats, no significant changes were detected in pituitary Pit-1, SST1, SST3 and GOAT mRNA levels versus the control groups (Fig. 3 & Suppl. Fig. S2 and S4); SST4 expression was not detectable (*data not shown*). Of note, the combination of both nutritional insults significantly reduced hypothalamic GHRH expression only in males (Fig. 4). SL/HFD males and females did not show alterations in hypothalamic SRIF and ghrelin expression versus SL/CD controls (Fig. 4). However, SL/HFD females exhibited a significant increase in hypothalamic NPY mRNA levels (Fig. 4).

## Discussion

The prevalence of obesity and its related metabolic disorders, such as type 2 diabetes and cardiovascular disease, has increased dramatically during the last decades worldwide, representing an enormous health and economic burden. Evidence from numerous studies has documented that nutritional alterations during early stages of development may contribute to enhance the obesity rates and the occurrence of obesity-related comorbidities^38,39^. Adverse metabolic conditions during the perinatal stage likely disrupt proper development of key neurohormonal systems due to the relative plasticity of some of their components during these early periods of development; phenomena that may have long-term consequences in energy homeostasis and modify the response to later metabolic challenges. In previous reports, we documented the impact of early postnatal overfeeding and HFD, alone or in combination, on metabolic and reproductive health in adult male and female rats^27,28^, with increased sensitivity to the deleterious impact of HFD being observed in animals overfed during the perinatal stage; a phenomenon also shown by others^29–31,40^. In the present study, we expand those studies by showing for the first time that perinatal overnutrition sensitizes also the GH axis for the detrimental effects of HFD-induced obesity; perturbations that may contribute to exacerbate the metabolic abnormalities linked to HFD consumption. Our study also documents striking sex-dependent differences in the regulatory mechanisms of the GH axis in obesity at the pituitary and hypothalamic level, which were associated with a differential impact of the combination of obesogenic insults on body length, which was overtly altered in SL/HFD males, but not in obese females.

It is widely accepted that there is a negative correlation between obesity and GH secretion. The deleterious impact of obesity on the somatotropic axis has been demonstrated in various species, from rodent to humans^12,13,16,21,41–43^. Of note, most of the studies addressing the detrimental impact of obesity on the GH axis have been conducted in males^14–16,21^, while females remained scarcely studied. In our current study, HFD alone caused rather modest alterations in the somatotropic axis in both sexes. This is possibly due to the relatively moderate nature of our HFD insult (45% fat content, causing a 10% elevation of BW) and the pulsatile nature of GH secretion^44^, which makes difficult to detect significant changes in mean values obtained from single blood samples. In any event, pituitary GH mRNA levels were not altered in HFD-induced obese rats of either sex, in keeping with previous reports showing similar pituitary GH content in lean and obese rats^45,46^. Furthermore, no other element of the somatotropic axis analyzed in HFD rats at adulthood was overtly altered, except for an increase in pituitary SST2 levels in females, suggesting that the impact, if any, of the HFD challenge alone on the GH axis is rather modest in our experimental model. In contrast, despite the fact that their metabolic profiles in adulthood were not as severely affected as in the HFD group, SL rats subjected to early postnatal overnutrition displayed durable alterations of several elements of the somatotropic axis, which were remarkably sexually different. These findings illustrate (1) the sensitivity of the GH axis to early nutritional challenges, which may have long-lasting consequences in somatotropic and related functions; and (2) the clear sex differences in the impact of such early nutritional challenges on the GH axis.

In addition, early onset overnutrition markedly sensitized the elements of the GH axis to the deleterious impact of obesity, in spite of the mild-to-null impact of HFD alone on various somatotropic parameters. Thus, SL/HFD males displayed suppressed GH mRNA expression in the pituitary, together with reduced hypothalamic GHRH and liver GH-R expression, while in SL/HFD females, a significant lowering of circulating GH and pituitary GH mRNA expression was observed, which was associated to suppressed expression of ghrelin at the pituitary and increased expression of NPY at the hypothalamus; the later might also contribute to the increased daily energy intake of SL/HFD versus control female rats. As none of these changes were detected in either SL or HFD rats, our data document the synergistic interaction of both obesogenic insults in perturbing the GH axis. Admittedly, the greater perturbation of the somatotropic axis in SL/HFD rats may derive, at least partially, from the higher severity of their metabolic perturbations, as compared to rats exposed to a single obesogenic insult (SL or HFD). However, considering that both male and female SL/HFD rats showed roughly similar alterations in key metabolic parameters, such as body weight gain, increased basal glucose levels and hyper-leptinemia, the observed sex differences in deregulated somatotropic factors are unlikely to be merely caused by this higher metabolic deterioration in SL/HFD animals, but rather represent a genuine phenomenon that documents the sexually-divergent mechanisms underlying the impact of early onset obesity on the GH axis.

The differential changes in the patterns of expression detected between male and female SL/HFD rats are likely indicative of distinct pathophysiological mechanisms for the perturbation of the hypothalamic and pituitary signals that govern GH output. Thus, while in SL/HFD males the suppression of pituitary GH mRNA expression may be driven by the inhibition of the hypothalamic expression of GHRH, as major physiological stimulus of GH synthesis and secretion^47^, in females, defective GH mRNA expression and circulating levels were not linked to suppressed GHRH expression at the hypothalamus, but rather to decreased pituitary expression of ghrelin and increased hypothalamic NPY mRNA levels. Lower ghrelin expression might be mechanistically relevant, as the pituitary ghrelin system has been reported to be an important local modulator of GH synthesis and release^48–50^. In addition, NPY has been suggested to negatively influence GH secretion in different experimental models and conditions^51,52^. Supporting this potential inhibitory effect of NPY on GH release, exogenous administration of NPY into the third ventricle has been shown to reduce plasma GH levels in ovariectomized rats^53^. Furthermore, in vitro studies have reported the negative effect of NPY on pituitary GH secretion^54^. Collectively, these findings suggest that the lowering of pituitary ghrelin and the rise in hypothalamic NPY expression detected in SL/HFD females might negatively affect pituitary GH expression.

In the same vein, other components of the somatotropic axis were affected by the combination of both obesogenic insults in a sexually divergent manner. Thus, SL/HFD males displayed increased expression of pituitary receptors for GH-stimulating signals, including GHRH-R and GHS-R, and decreased expression of SST2, the main inhibitory receptor subtype involved in the GH-inhibiting actions of SRIF. These pituitary changes may suggest a compensatory mechanism of the somatotropes in response to reduced GH levels in this experimental group. In contrast, the gene expression profiles of the receptors for GH-stimulating and -inhibiting signals were unaltered in SL/HFD females. In addition, while circulating IGF-1 and hepatic expression of IGF-1 gene, whose levels have been correlated with changes in circulating GH in various experimental models^55^, were not altered in SL/HFD rats of either sex, liver expression of GH-R was significantly reduced in males, but not females exposed to both obesogenic insults. This would suggest an impaired hepatic sensitivity to GH particularly in males. Given the well-known actions of GH in the liver for proper glucose homeostasis^56^, the contribution of such alteration upon the systemic metabolic perturbations seen in obese male rats merits further investigation. Interestingly, hepatic insulin resistance has been previously proposed as an early metabolic defect in states of severe GH deficiency^57^.

Compelling evidence suggests that somatotropes may play a role as metabolic sensors of the organism^58–60^. Various metabolic hormones, such as leptin and insulin, have been reported to regulate somatotrope function and, consequently, GH secretion, which in turn would influence body composition^59,61^. Selective deletion of pituitary leptin receptors in mice has been associated with GH deficiency and development of obesity^59,62^; such an effect was more pronounced in males^62^. In our study, a clear sexual difference was found regarding the pituitary expression of leptin receptor mRNA, whose levels were significantly up-regulated only in SL/HFD males. Given the positive effects of leptin on pituitary GH, this might suggest the existence of a compensatory mechanism in response to decreased GH mRNA levels. On the other hand, pituitary insulin receptor expression was also enhanced in SL/HFD males, but not females. Insulin carries out inhibitory actions on pituitary GH synthesis and secretion^21,61^. Although no significant differences in circulating insulin levels were detected between SL and SL/HFD males, such an increase in pituitary insulin receptor expression, and therefore in pituitary insulin sensitivity, might also contribute to the reduction in pituitary GH mRNA levels observed in SL/HFD males.

The question arising from our data is what are the potential mechanisms underlying the sexually different impact of early obesity on the somatotropic axis. While these were not directly addressed by our current study, it is tenable to speculate that epigenetic processes, taking place during early stages of development, might differentially affect gene expression of key elements of the GH axis in males and females, which may predispose to sex-specific responses to HFD at later developmental stages^26,29–31^. In addition, sex-specific changes in gut microbiota composition induced by early onset obesity may also contribute, via epigenetic mechanisms, to disrupt proper adaptation to nutritional insults later in life^63^, which may compromise the correct functioning of the somatotropic axis in the long-term. Finally, changes in sex steroids might also contribute to the observed sexually-divergent patterns. It is known that sex hormones can modulate GH output and actions, and alter GH signaling pathways in peripheral tissues^64^; alterations in sex steroid levels and gonadal function are bound to obesity, with a differential impact between males and females^27,28^. Hence, sex-specific obesity-related changes in gonadal steroid levels might alter also the gene expression of certain elements of the somatotropic axis, thus promoting sex differences.

**Figure 5:**
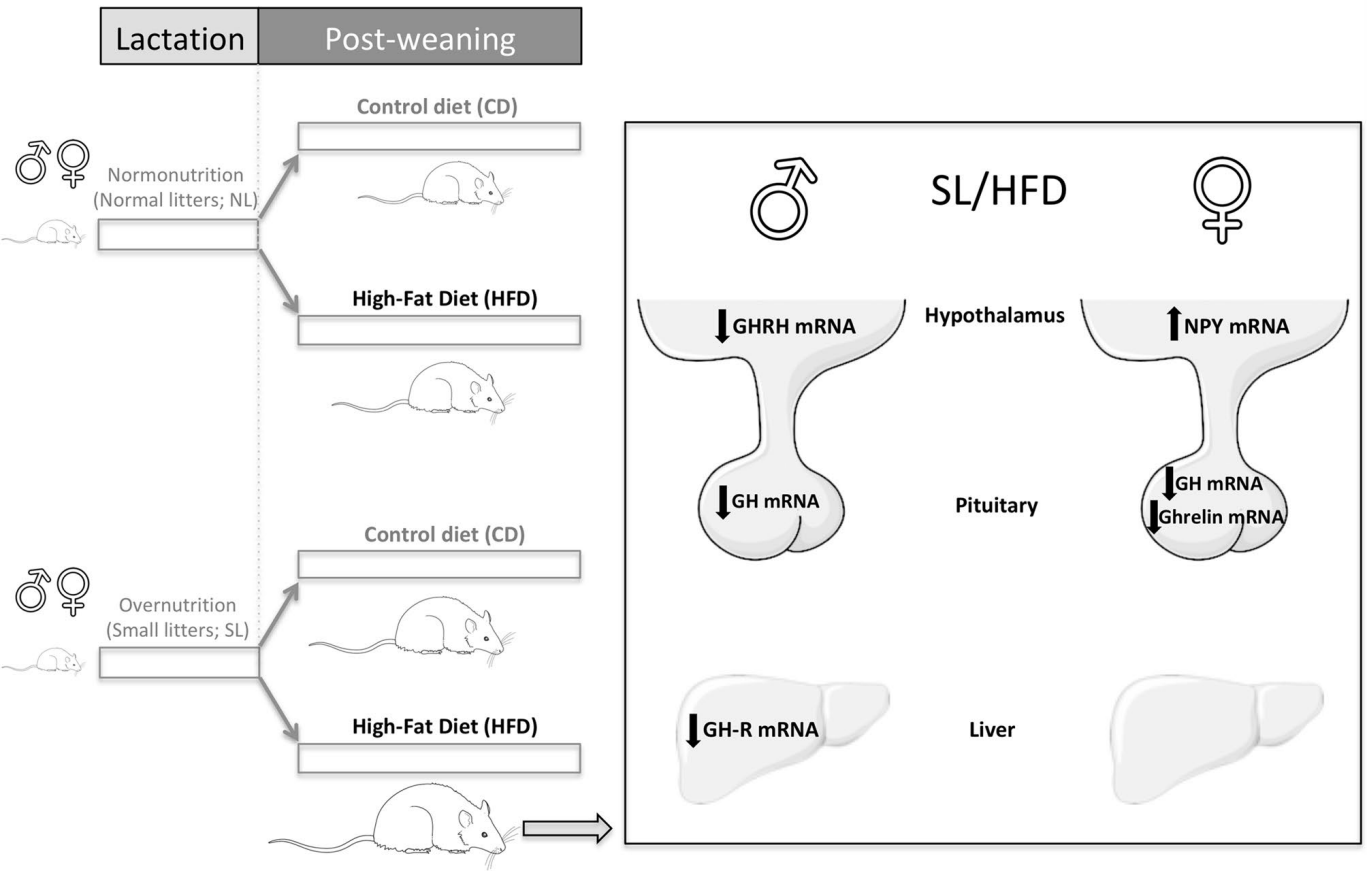
Graphical summary of the most salient alterations in key elements of the GH axis caused by the obesogenic insults, SL (rearing in small litters) and HFD (exposure to high fat content diet from weaning onwards), in male and female rats, with particular focus on the impact of combined exposure to these obesogenic insults and sexually-divergent perturbations. The figure was created by the authors, using free medical images obtained from Servier Medical Art (https://smart.servier.com).

In sum, our findings demonstrate that exposure to an obesogenic nutritional environment during early periods of development not only enhanced the metabolic perturbations caused by HFD, but also exacerbated the detrimental effects of HFD-induced obesity on the somatotropic axis in both sexes. Yet, our data also disclose clear discrepancies between males and females concerning the neurohormonal mechanisms that deregulate the GH axis in obesity conditions due to the combination of early overnutrition and HFD. Whereas in males the decline in pituitary GH expression is mostly attributed to decreased hypothalamic GHRH expression, in females, pituitary down-regulation of ghrelin expression along with increased hypothalamic expression of NPY may be key mechanisms responsible for the suppression of the GH axis (see Fig. 5). Pending additional, confirmatory measurements of the actual changes of tissue protein levels and time-course analyses of putative alterations of the elements of the GH axis after exposure to obesogenic insults at various developmental windows, our present study, addressing mainly changes in hormonal and gene expression levels, highlights the importance of early nutritional programming as a contributing factor for the vulnerability of the GH axis to HFD, thus suggesting that the early nutritional environment may be an important modifier of the GH axis in adulthood in conditions of diet-induced obesity.

## Materials and methods

### Animals and diets

Wistar male and female rats bred in the vivarium of the University of Córdoba were used. Animals were maintained under constant conditions of light (14 h of light, from 07:00 h) and temperature (22 °C), with free access to pelleted food and tap water. Animals were fed a control diet [CD; D12450B: 10% of calories from fat, 20% from protein, and 70% from carbohydrate], or a high-fat diet [HFD; D12451: 45% of calories from fat, 20% from protein, and 35% from carbohydrate; Research Diets Inc.]. Experimental procedures were approved by Córdoba University Ethical Committee and conducted in accordance with European Union guidelines.

### Experimental design

In order to explore whether early postnatal overnutrition may affect the somatotropic axis and alter its sensitivity to the deleterious effects of HFD in adult male and female rats, expression and hormonal analyses were implemented in serum and tissue samples from cohorts of male and female rats, generated in the context of large longitudinal studies addressing the impact of these sequential obesogenic insults on various reproductive and metabolic parameters^27,28^. As model of early postnatal overnutrition, we used a well-established experimental protocol of litter size manipulation during the lactation period, which has been widely used by our numerous groups, including ours, to induce conditions of postnatal overfeeding^27–31,65,66^. Pregnant dams were obtained by mating with adult males of proven fertility. On postnatal day (PND) 1, male and female pups were selected, cross-fostered, and grouped into two different litter sizes: normal litters (NL; 12 pups per litter) and small litters (SL; four pups per litter) in order to induce early postnatal normo- and over-nutrition, respectively. On PND 23, the animals were weaned and pups from each litter size were housed in groups of four rats per cage. From weaning onward, the animals were fed either a CD or a HFD ad libitum. This experimental design allowed us to generate four experimental groups in both males and females: NL/CD, NL/HFD, SL/CD and SL/HFD. One week before the animals were euthanized, daily energy intake was calculated from mean food ingestion per day (during 1-wk) using the kcal/g index provided by the manufacturer (3.85 kcal/g for CD; 4.73 kcal/g for HFD). In the morning of PND 120, all animals (males and females) from the different experimental groups were weighed and humanely killed by decapitation and trunk blood, pituitary, hypothalamus, and liver were collected for further analysis. An overview of the experimental design is represented in Suppl. Fig. S1.

### Hormonal measurements

After euthanasia, trunk blood was collected from all animals and centrifuged at 3.000 rpm for 20 min at 4 °C. Serum was stored at − 80 °C until evaluation of GH, IGF-1, leptin and insulin levels. GH was measured using a rat ELISA kit (Millipore Corporation; Billerica, MA). The sensitivity of the assay was 0.07 ng/mL, and the intra- and inter-assay coefficients of variation (CV) were 1.7 and 4.9, respectively. Serum IGF-1 levels were also determined using a commercial ELISA kit (IDS; Boldon, UK). In this case, the sensitivity was 63 ng/mL and the intra- and inter-assay CV were 4.3 and 6.3, respectively. Serum leptin and insulin levels were measured using RIA kits from Linco Research (St. Charles, MO); the sensitivity of the assays was 0.5 (leptin) and 0.1 (insulin) ng/mL.

### RNA isolation and retro-transcription (RT)

After decapitation of the animals, whole hypothalami, pituitaries, and liver extracts were immediately collected, frozen in liquid nitrogen and stored at − 80 °C until used for RNA isolation. Tissues were processed for recovery of total RNA using the Absolutely RNA RT-PCR Miniprep Kit (Stratagene; La Jolla, CA), with deoxyribonuclease treatment. Total RNA concentration and purification was assessed by using a NanoDrop-2000 spectrophotometer (Thermo Scientific; Waltham, MA). For the retro-transcription, 1 μg of RNA was reversed transcribed using random hexamer primers, with enzyme and buffers supplied in the First Strand cDNA Synthesis kit (MRI Fermentas; Hanover, MD). Duplicate aliquots were amplified by quantitative real time PCR, in which samples were run against specific synthetic standards of each transcript analyzed to estimate mRNA copy number (*see below*).

### Quantitative real-time RT-PCR

Analyses were conducted following previously published protocols^21^. Primers sets (see Table 1) were designed using Primer 3 software (Rosen, S., and H. J. Skaletsky, 2000; Whitehead Institute for Biomedical Research), using rat genomic sequences as templates. PCR assays were conducted with the 2X Master Mix PCR reagent (MRI Fermentas; Hanover, MD), using specific primer pairs and cDNA from RT reactions as template. Profiles for PCR amplification were as follows: one cycle of 95 °C for 10 min, followed by 35 cycles of 95 °C for 1 min, 61 °C for 1 min, and 72 °C for 1 min; a final cycle of 72 °C for 10 min was included. PCR products were run on agarose gels for visual verification under ethidium bromide staining, and subjected to column purification (QIAGEN, Valencia, CA) and sequencing to confirm amplicon specificity.

**Table 1:** Specific sequence of primers used in the study for quantitative real-time PCR and product sizes.

Primer	Sequence		Product size (bp)
*GH*	5′-TTA CCT GCC ATG CCC TTG T-3’ 5′-TGT AGG CAC GCT CGA ACT CT-3’	Sense Antisense	106
*GHRH-R*	5′-AGT CCT CTC TGT TGG GGT GAA-3’ 5′-ACA GCG GGA TAA GGA GAA GTG-3’	Sense Antisense	146
*SST1*	5′-TGC CCT TTC TGG TCA CTT CC-3’ 5′-AGC GGT CCA CAC TAA GCA CA-3’	Sense Antisense	134
*SST2*	5′-CCC ATC CTG TAC GCC TTC TT-3’ 5′-GTC TCA TTC AGC CGG GAT TT-3’	Sense Antisense	134
*SST3*	5′-TTG GCC TCT ACT TCC TGG TG-3’ 5′-ATC CTC CTC CTC CTC CGT CT-3’	Sense Antisense	199
*SST4*	5′-ACA ACT TCC GAC GCT CTT TC-3’ 5′-CTC TTC CTC AGC ACC TCC A-3’	Sense Antisense	199
*SST5*	5′-TCA TTG TGG TCA AGG TGA AGG-3’ 5′-AAG AAA TAG AGG CCG GCA GA-3’	Sense Antisense	199
*Ghrelin*	5′-TCC AAG AAG CCA CCA GCT AA-3’ 5′-AAC ATC GAA GGG AGC ATT GA-3’	Sense Antisense	126
*Pit-1*	5′-GGA AGA GGA AAC GGA GGA CA-3’ 5′-TCG GTT GCA GAA CCA CAC TC-3’	Sense Antisense	158
*Insulin-R*	5′-TCA TGG ATG GAG GCT ATC TGG-3’ 5′-CCT TGA GCA GGT TGA CGA TTT-3’	Sense Antisense	129
*Leptin-R*	5′-GGA AGG AGT TGG AAA ACC AAA G-3’ 5′-TCC GAG CAG TAG GAC ACA AGA-3’	Sense Antisense	127
*GHRH*	5′-TGG GTG TTC TTT GTG CTC CT-3’ 5′-CTT TGT TCC TGG TTC CTC TCC-3’	Sense Antisense	197
*SRIF*	5′-TCT GCA TCG TCC TGG CTT T-3’ 5′-CTT GGC CAG TTC CTG TTT CC-3’	Sense Antisense	113
*NPY*	5′-TCG CTC TAT CCC TGC TCG T-3’ 5′-GGG GCA TTT TCT GTG CTT TC-3’	Sense Antisense	220
*GH-R*	5′-ACT GGC AGC ATG TGA AGA AGA-3’ 5′-GGA ACT GGT ACT GGG GGT AAA-3’	Sense Antisense	147
*IGF-1*	5′-TTG TGG ATG AGT GTT GCT TCC-3’ 5′-GGT CTT GTT TCC TGC ACT TCC-3’	Sense Antisense	179
*GOAT*	5′-ATT TGT GAA GGG AAG GTG GAG-3’ 5′-CAG GAG AGC AGG GAA AAA GAG-3’	Sense Antisense	120
*Cyclophilin-A*	5′-TGG TCT TTG GGA AGG TGA AAG-3’ 5′-TGT CCA CAG TCG GAG ATG GT-3’	Sense Antisense	97
*β-actin*	5′-CCT AAG GCC AAC CGT GAA A-3’ 5′-CCA GAG GCA TAC AGG GAC AA-3’	Sense Antisense	104
*Hprt*	5′-AGC TTG CTG GTG AAA AGG AC-3’ 5′-TCC ACT TTC GCT GAT GAC AC-3’	Sense Antisense	153

*GH* growth hormone; *GHRH-R* GH-releasing hormone-receptor; *SST1, SST2 SST3, SST4* and *SST5* somatostatin receptor 1, 2, 3, 4 and 5; *GHRH* GH-releasing hormone; *SRIF* somatostatin; *NPY* neuropeptide Y; *GH-R* growth hormone receptor; *IGF-1* insulin-like growth factor-1; *GOAT* ghrelin-O-acyltransferase; *Hprt* hypoxanthine phosphoribosyl-transferase

Quantitative real-time PCRs (qPCR) were performed using the qPCR Stratagene Mx3000p instrument (Agilent, Santa Clara, CA, USA) with Brilliant III SYBR Green Master Mix (Agilent). The development, validation and application of the qPCR to measure the expression levels of different transcripts have been previously reported^21^. Briefly, absolute gene expression levels were calculated using a specific standard curve for each transcript analyzed (1, 10^1^, 10^2^, 10^3^, 10^4^, 10^5^, and 10^6^ copies of synthetic template). A No-RT sample was used as a negative control. For each qPCR reaction, 10 μl of master mix, 0,3 μl of each primer (10 μM stock), 8,4 μl of distilled H_2_O and 1 μl of cDNA (50 ng) were mixed with a program consisting of the following steps: (1) 95 °C for 3 min, (2) 40 cycles of denaturing (95 °C for 20 s) and annealing/extension (61 °C for 20 s) and, (3) a graded temperature-dependent dissociation step (55 °C to 95 °C, increasing 0.5 °C/30 s) to verify that only one product was amplified. To control for variations in the amount of RNA used and the efficiency of the RT-reaction, the expression level of each transcript was adjusted by a Normalization Factor (NF) in each sample obtained from the expression levels of three housekeeping genes (*β-actin*, *Hprt* and C*yclophilin A*) using the Genorm program. Expression level of the housekeeping genes analyzed did not differ between experimental groups (data not shown).

### Data presentation and statistical analysis

As mentioned above, experiments addressing the effects of early postnatal overnutrition on absolute mRNA copy number of the transcript of interest within the whole pituitary, hypothalamic and hepatic extracts were adjusted by a NF in each sample using the absolute mRNA copy number of three housekeeping genes. RNA analyses were performed in duplicate from at least 5 independent samples per group, in line with previous studies of the group addressing pituitary changes in mRNAs expression levels of GH-related factors^21,36^. The total number of animals included in each experimental group and the minimal numbers of independent samples (n = 8) for hormonal determinations in the study are shown in Fig. 1. All data are expressed as the mean ± SEM. For the analysis of the effects of the two major variables under study (litter size and diet) and their interactions, two-way analysis of variance (ANOVA) was performed. Significance level was set at *P* ≤ 0.05 (GraphPad Software Inc., La Jolla, CA). When significant differences were found, the data were further analyzed through post hoc comparison using Newman–Keuls tests to identify simple effects, in keeping with previous studies^27,28^. Considering the inherent variability of the factors under analysis, when *P* values ranged between < 0.1 and > 0.05, a trend for significance was indicated, where appropriate.

## Supplementary information

Supplementary information.

## Data Availability

The data supporting the findings and conclusions of this study are included in this article and its supplementary files. All relevant original data are available from the corresponding authors, upon reasonable request.
