# Supplementary material for: Early overnutrition sensitizes the growth hormone axis to the impact of diet-induced obesity via sex-divergent mechanisms
Source: Sci Rep. 2020 Aug 17;10:13898. doi: 10.1038/s41598-020-70898-y (PMC7431568; doi:10.1038/s41598-020-70898-y)
Supplement: Supplementary file 1 — Supplementary information. [file 41598_2020_70898_MOESM1_ESM.pdf]

SUPPLEMENTAL MATERIAL

Early Overnutrition Sensitizes the Growth Hormone Axis to the Impact of Diet-Induced Obesity via sex-divergent mechanisms

M.A. Sanchez-Garrido[1,2], Francisco Ruiz-Pino[1,2], A.I. Pozo-Salas[1,2], J.M. Castellano[1,2,3,4], M.J. Vazquez[1,2], R.M. Luque[1,2,3,4¶], M. Tena-Sempere[1,2,3,4,5¶]

[1]Maimónides Institute of Biomedical Research of Córdoba (IMIBIC), Cordoba, Spain; [2]Department of Cell Biology, Physiology & Immunology, University of Córdoba, Córdoba, Spain; [3]Reina Sofia University Hospital, Córdoba, Spain; [4]CIBER Fisiopatología de la Obesidad y Nutrición (CIBEROBN), Instituto de Salud Carlos III, 14004 Córdoba, Spain; and [5]Institute of Biomedicine, University of Turku, 20520 Turku, Finland

¶ Equal senior authors

Running Title: Obesity and the GH Axis

Key Words: Early overnutrition, obesity, growth hormone (GH), somatotropic axis, metabolism

Corresponding author: Raul M. Luque (bc2luhur@uco.es)
Manuel Tena-Sempere (fi1tesem@uco.es)
Department of Cell Biology, Physiology & Immunology
Faculty of Medicine, University of Córdoba
Avda. Menéndez Pidal s/n. 14004 Córdoba, SPAIN

Number of Pages: 25 pages

Number of Figures and Tables: 5 figures (+ 4 Supplemental) + 1 table

Word count: 4,445 (Main Text; Title, Abstract, References and Figure Legends excluded)

Conflict-of-Interest Statement: The authors declare no conflict of interest in relation with the contents of this article

Supplemental Figures

Supplemental Figure S1. Schematic representation of the nutritional manipulations and experimental groups included in the study. Nutritional insults were applied in different stages of development in order to generate the different experimental groups. Numbers of animals included in each experimental group, and the minimal numbers of independent samples for hormonal determinations in the study are also shown.

Supplemental Figure S2. Pituitary SST1, SST3, leptin and insulin receptor mRNA levels in male (*left panels*) and female rats (*right panels*) subjected or not to overfeeding during lactation (SL vs. NL) and/or HFD after weaning (HFD vs. CD). mRNA copy numbers were determined by qPCR and were adjusted by a Normalization Factor (NF) in each sample obtained from the expression levels of three housekeeping genes (*β-actin*, *Hprt* and C*yclophilin A*). Data are presented as mean ± standard error of the mean, n = 8 per experimental group. Statistically significant differences were assessed by two-way ANOVA to analyze the effects of litter size and diet and their interactions. When significant differences were found, the data were further analyzed using Newman-Keuls tests to identify simple effects. *, $P < .05$; **, $P < .01$ effect of HFD; a, $P < .05$, effect of litter size; b, $P < .05$ interaction of litter size/HFD.

Supplemental Figure S3. Circulating levels of glucose, insulin and leptin and energy intake in male and female rats subjected or not to overfeeding during lactation (SL vs. NL) and/or HFD after weaning (HFD vs. CD). Metabolic characterization of these models was partially reported in our previous publications [27, 28]. Data are presented as mean ± standard error of the mean (SEM), n = 8 per experimental group. Statistically significant differences were assessed by two-way ANOVA to analyze the effects of litter size and diet and their interactions. When significant differences were found, the data were further analyzed using Newman-Keuls tests to identify simple effects. *, $P < .05$; **, $P < .01$ effect of HFD; a, $P < .05$, effect of litter size; b, $P < .05$ interaction of litter size/HFD.

Supplemental Figure S4. Pituitary GOAT mRNA levels and body size (naso-anal length) in male (*left panels*) and female rats (*right panels*) subjected or not to overfeeding during lactation (SL vs. NL) and/or HFD after weaning (HFD vs. CD). For the analysis of pituitary GOAT expression, mRNA copy numbers were determined by qPCR and were adjusted by a Normalization Factor (NF) in each sample obtained from the expression levels of three housekeeping genes (*β-actin*, *Hprt* and C*yclophilin A*).

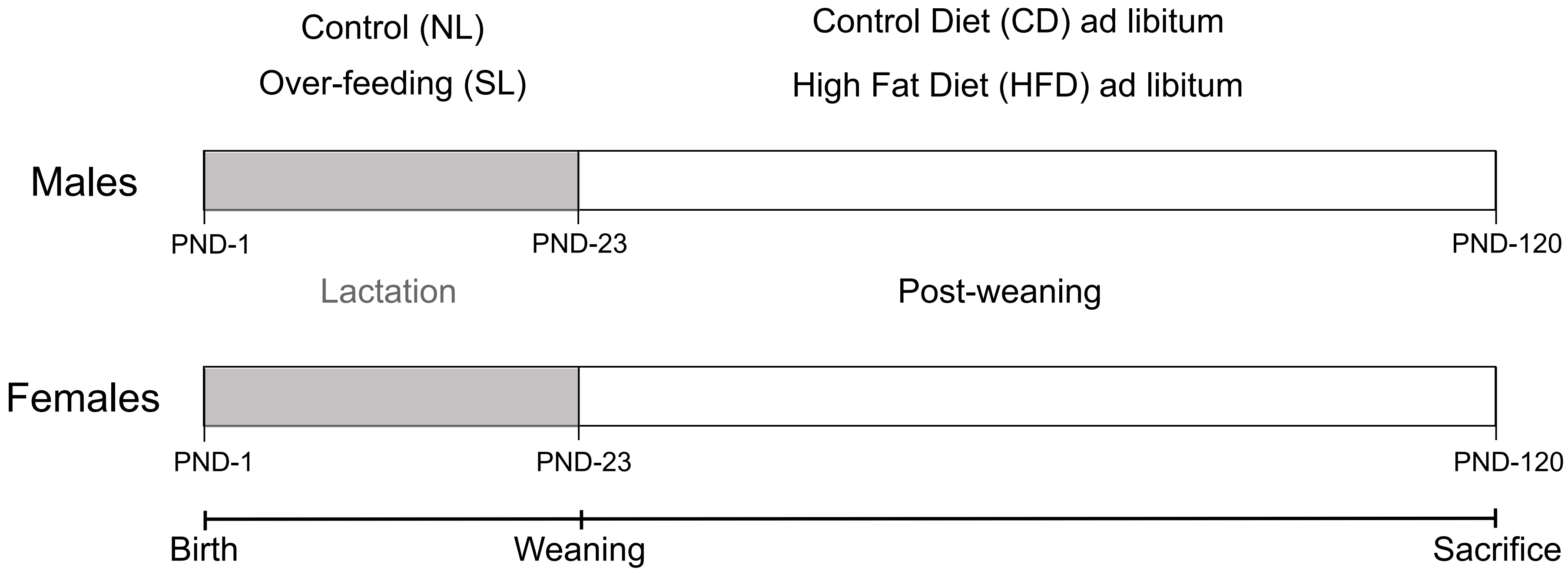

Group	Description	Males (n)	Females (n)	Hormonal determinations Males & Females (n)
NL/CD	Normal litter & control diet: Control group	20	24	8
NL/HFD	Normal litter & high fat diet post-weaning	15	24	8
SL/CD	Small litter & control diet: Effect of postnatal over-feeding	18	17	8
SL/HFD	Small litter & high fat diet: Effect of postnatal over-feeding + HFD	16	20	8

Supplemental Figure S1

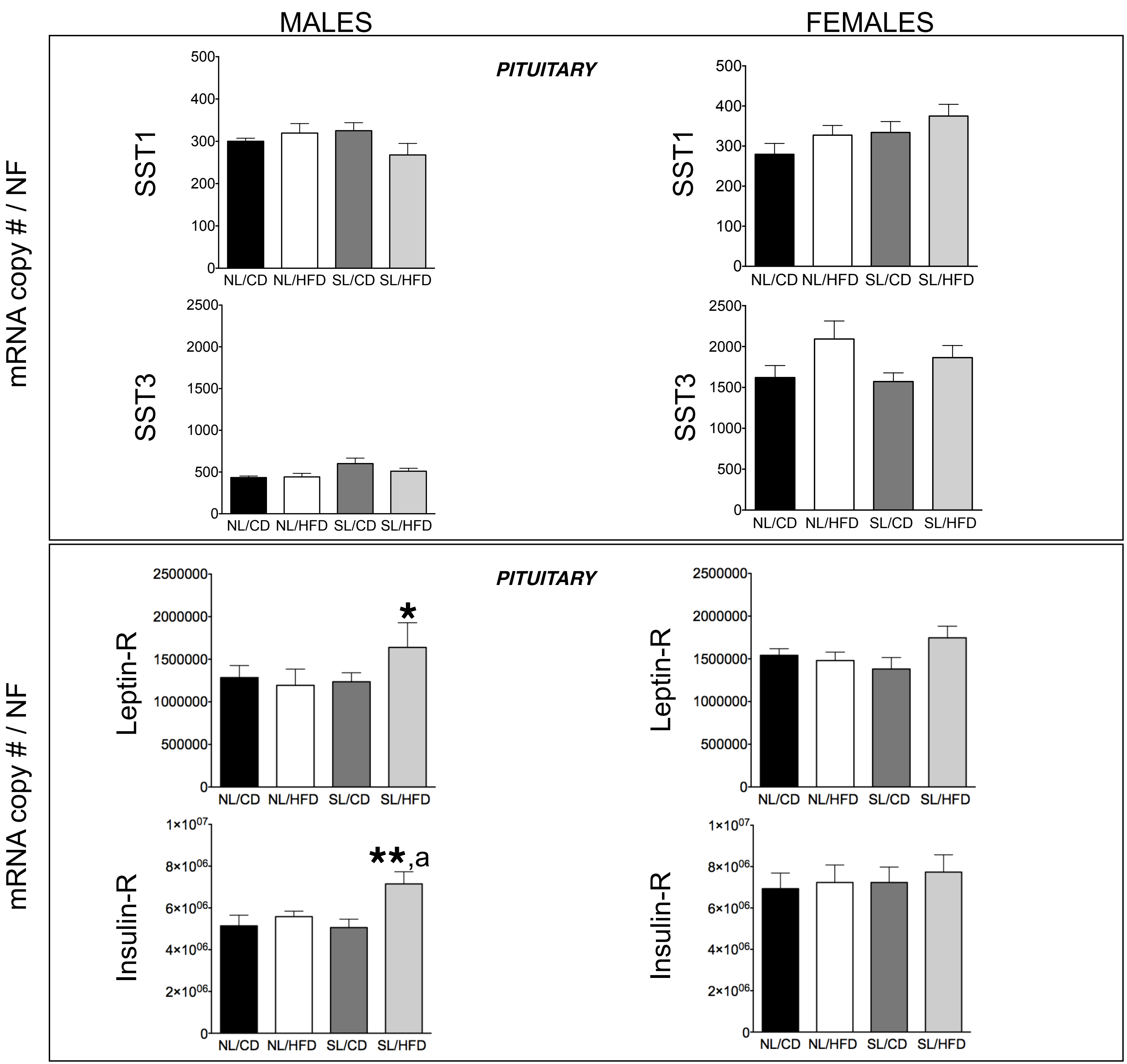

Supplemental Figure S2

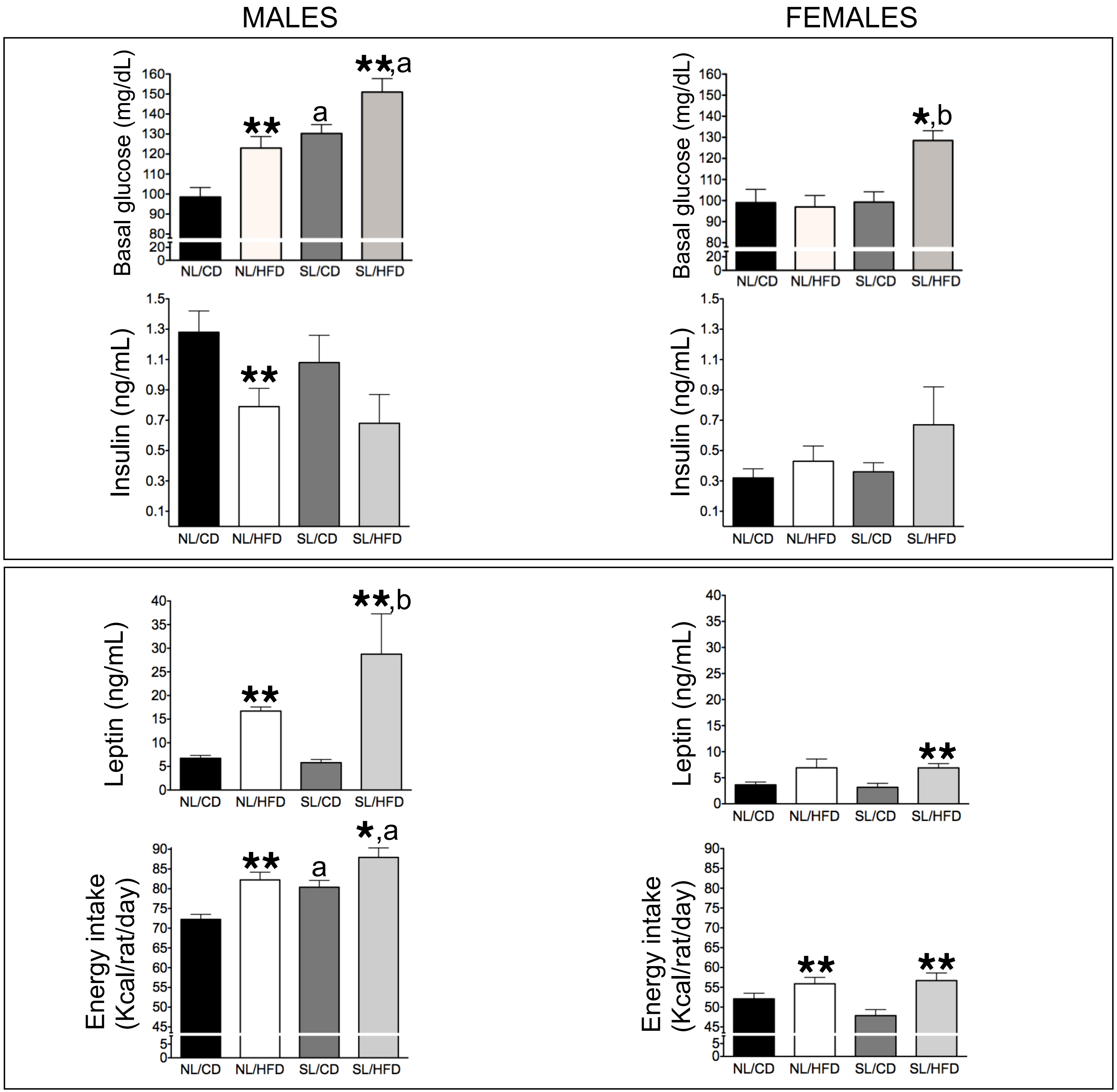

Supplemental Figure S3

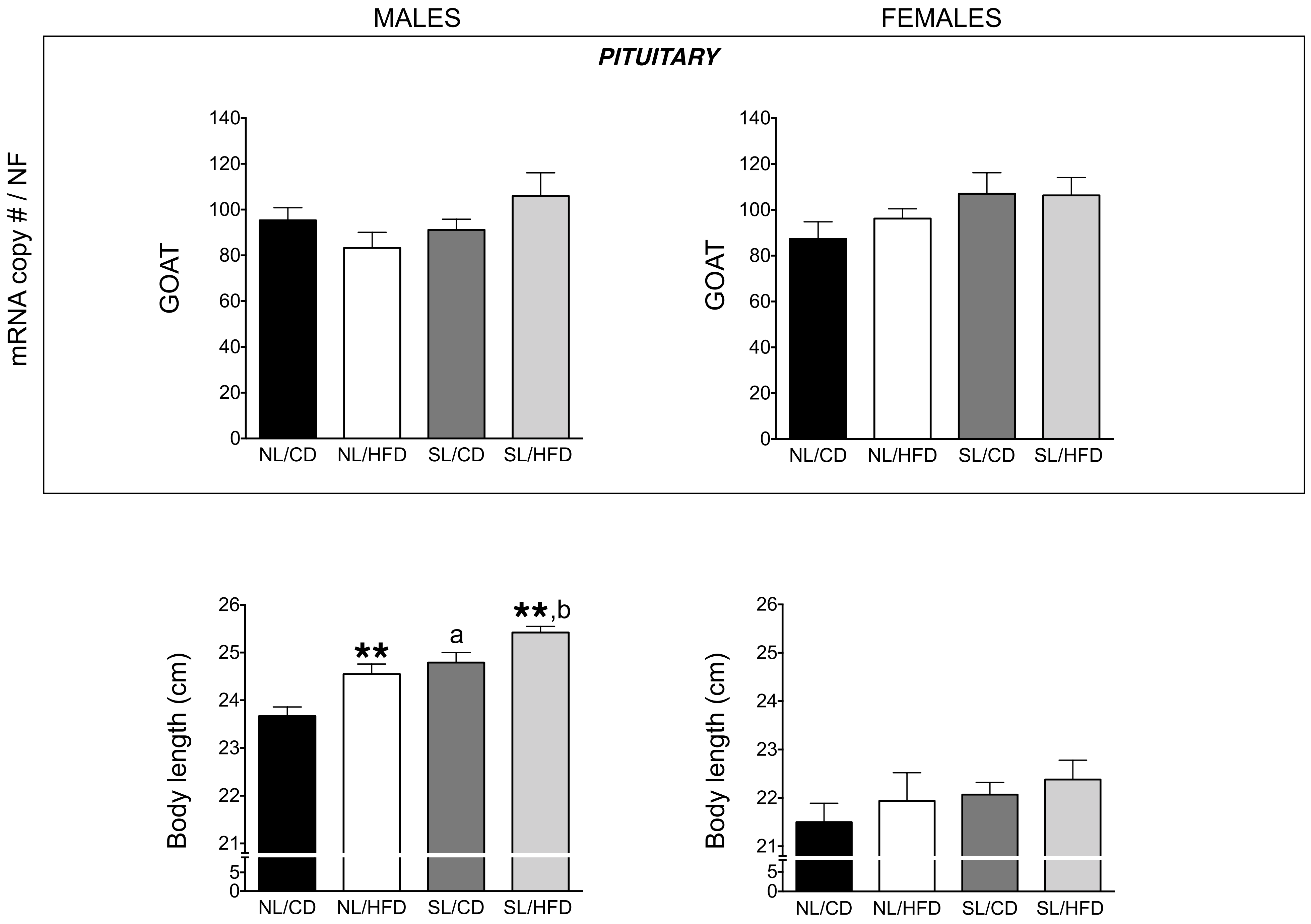

Supplemental
Figure S4
